# Glucose Determination by a Single 1535 nm Pulsed Photoacoustic Technique: A Multiple Calibration for the External Factors

**DOI:** 10.1155/2022/9593843

**Published:** 2022-09-19

**Authors:** Lifeng Yang, Chulin Chen, Zhaojiang Zhang, Xin Wei

**Affiliations:** School of Optoelectronic Science and Engineering, University of Electronic Science and Technology of China, Chengdu 611731, China

## Abstract

Photoacoustic spectroscopy has been proved to be a potential method for noninvasive blood glucose detection. We used 1535 nm pulsed laser to excite photoacoustic signal in glucose solution and then explored the influence of different glucose concentration on photoacoustic signal to analyze the sensitivity of photoacoustic signal to glucose at this wavelength. We designed a simple photoacoustic cell structure, which used a focused ultrasonic transducer to receive signals, so as to reduce signal attenuation. In terms of the results, we have found that for high-concentration glucose solutions, the results have strong linearity and discrimination, and when the concentration is close to the human body level, the signal difference is not so obvious. Therefore, we explore the external factors affecting the photoacoustic signal in detail and propose a calibration method. Through calibration, the signal generated by the low-concentration glucose solution also has a good linearity.

## 1. Introduction

Diabetes is a common metabolic disease that can lead to serious complications, such as cardiovascular disease, stroke, blindness, chronic renal failure, neuropathy, and even death [1]. According to the latest data from the ninth edition of the IDF Diabetes Atlas, as of 2019, a total of 463 million adults worldwide have diabetes and one out of every 11 adults aged 20–79 will have diabetes [2]. Most current glucose meters require a finger prick to collect blood samples for testing, which cannot provide continuous glucose monitoring (CGM) and have a risk of wound infection. For this reason, there is an urgent need for a noninvasive monitoring of blood glucose while photoacoustic spectroscopy is one of the potential solutions. Compared with traditional spectroscopic techniques, photoacoustic spectroscopy collects the voiceprint signal generated by light irradiation, effectively avoiding the error caused by reflection, refraction, and scattering in tissue [3].

Noninvasive blood glucose monitoring within photoacoustic has been studied for nearly 30 years since 1993 [4]. And then, Mackenzie et al. [5, 6], Bednov et al. [7, 8], Kinnunen and Myllylä [9], von Lilienfeld-Toal et al. [10] have demonstrated the feasibility of this technique. In the past 10 years, there have been three main types of noninvasive blood glucose detection using photoacoustic spectroscopy. The first is the mid-infrared (MIR) pulse schemes represented by Pleitez et al. [11–13] and Kottmann et al. [14, 15]. In these schemes, the MIR pulsed laser is used and the signal is detected in the air with a microphone. The schemes have been tested in vivo in 2013 and have achieved some results [13], but due to the high cost of laser, there has not been much research progress in recent years. Another is the near-infrared (NIR) pulse schemes represented by Pai et al. [16–18], Zhang et al. [19–21], and Zhao et al. [22, 23]. In these schemes, the NIR pulsed lasers are used, and the effective wavelengths are mainly concentrated around three wavelengths of 905 nm, 1064 nm, and 1600 nm, or use full-wavelength scanning, and a microphone or ultrasonic transducer is used to detect the signal. Generally, the ultrasonic transducer will be contact with the absorbing medium directly. Most of the researches in recent years were focused on these schemes. The other is NIR continuous wave laser schemes represented by Camou et al. [24, 25] and Tanaka et al. [26, 27]. These schemes are mainly led by members of NTT Device Technology Labs in Japan. They hoped to use continuous laser to generate signals instead of pulsed lasers. It has taken nearly 10 years from the initial proposal to the in vivo experiment, and there are still some works to be tried [27].

In recent years of research, for the scheme using single-wavelength lasers, in order to obtain obvious linearity, a very high concentration of glucose solution was usually used, which was several orders of magnitude higher than human body level. It has usually reached 2000 mg/dL [14, 19, 25]. To make the concentration accurate to the level of the human body, multiwavelength calibration or full-wavelength scanning was commonly used; otherwise, complex structures and algorithms were required, which increased the cost and redundancy of the system [13, 20, 26].

A new study for blood glucose measurement usually starts with glucose solution then in vitro and in vivo [19, 22, 24]. We designed an experimental device with a simple structure, using a 1535 nm single-wavelength pulsed laser to explore the influence of different glucose concentrations on the photoacoustic signal and the errors caused by external factors. In this paper, the errors caused by laser energy, temperature, and sample structure in glucose solution experiment were studied in detail. Then, by using multivariate feature calibration, we improved the linearity of photoacoustic signal within the single-wavelength method in human blood glucose levels.

## 2. Photoacoustic Methods

Laser irradiation on the absorbing medium will produce an ultrasonic wave. This ultrasonic wave is called a photoacoustic signal. The pressure generated in the photoacoustic process can be described by the wave equation as follows [28]:

(1) $$\begin{array}{c} \left[\frac{1}{v^{2}}\frac{\partial^{2}}{\partial t^{2}}-\nabla^{2}\right]p=\frac{\alpha \beta }{C_{p}}\frac{\partial I}{\partial t}\text{,} \end{array}$$

where *I* is light intensity, *V* is sound velocity in the medium, *α* is the optical absorption coefficients, *β* is the coefficient of thermal expansion, *C*_*p*_ is the specific heat capacity, and *p* is sound pressure. When the absorption of the sample is weak, the peak pressure *p* can be expressed as follows [29]:

(2) $$\begin{array}{c} p=k\frac{\beta \sqrt{v}}{C_{p}}E_{0}\alpha \text{.} \end{array}$$

*k* is the system constant and *E*_0_ is incident laser energy; thus,

(3) $$\begin{array}{c} p\propto \frac{\alpha \beta \sqrt{v}}{C_{p}}\text{.} \end{array}$$

When other parameters are constant, the peak pressure is proportional to the coefficient of optical absorption *α* and the coefficient of thermal expansion *β*. And changing glucose concentration will affect the optical properties of blood, thereby changing various coefficients. Thus, the change of pressure peak can indirectly respond the change of glucose concentration.

## 3. Experimental Setup

**Figure 1:**
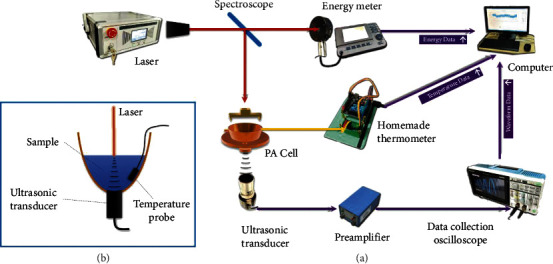
The overview of the system. (a) The experiment system. (b) Structure of PA cell.

The experimental equipment is shown in Figure 1, which is mainly composed of the laser, energy meter, thermometer, ultrasonic transducer, photoacoustic cell (PA Cell), preamplifier, data collection oscilloscope, and computer. We use a semiconductor pulsed laser (Model JT-EGML-E400F-10) as the light source, with a center wavelength of 1535 nm, a pulse width of 4 ns, and a single pulse energy of 365 ± 2 *μ*J. The ultrasonic transducer is the type of V309-SU-F-1.20-IN-PTF from Olympus, and its center frequency is 2.25 MHz. The preamplifier (Model 5678 PREAMP, Panametrics-NDT, Olympus, USA) can amplify the signal by 30 dB. The thermometer is homemade with the resolution of 0.01°C.

The laser delivered pulses laser at a pulse repetition rate (PRR) of 4 Hz. Through the spectroscope, 30% of the energy is transferred to the energy meter. The energy meter transmits the detected value to the computer for subsequent processing. The remaining 70% of the laser is conveyed to the PA cell. The PA cell is a bowl-shaped structure, the ultrasonic transducer is fixed at the bottom, and the sample is stored on the upper part to submerge the transducer. The transducer is water-immersed focusing type. After adding 25 mL of the liquid into the PA cell, the focus of the transducer will fall near the liquid surface and the laser will be irradiated on here. Since the high absorption by water, the light is already absorbed before it reaches the transducer, so there is no need to worry about the impact of the pulsed laser on the ultrasonic transducer. When laser is irradiated on the surface of the liquid, a photoacoustic signal is generated. The photoacoustic signal (PA signal) is an ultrasonic wave, which is different from the optical signal in that its loss in the liquid is very small. The signal is received by ultrasonic transducer and then amplified by preamplifier. After that, it is transmitted to the oscilloscope, which can collect data and can be connected to a computer. A temperature probe is immersed under the sample and the thermometer can communicate with the computer to transmit temperature data in real time. In this experiment, the laser emitter, spectroscope, and ultrasonic transducer are all firmly fixed, which prevents large errors caused by the relative displacement.

## 4. Results

### 4.1. Direct Measurement of the PA Signal

**Figure 2:**
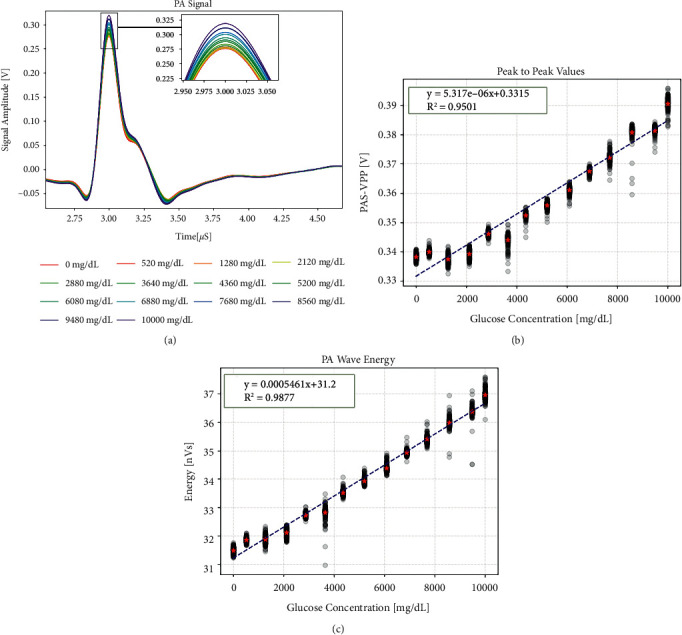
Photoacoustic signal difference in high-concentration glucose solution. (a) Waveform difference. (b) Peak-to-peak difference. (c) Waveform energy difference.

In order to verify that changes in glucose concentration can indeed affect the PA signal, we first used a high concentration of glucose solution for experiments. The laser was preheated for 20 minutes before the experiment to keep the laser energy constant, and the whole experiment was performed at room temperature. The glucose concentration ranges from 0 to 10000 mg/dL and increases by a value around 500 mg/dL. The order of concentration was disarranged to prevent the influence of external factors which vary linearly over time. For each concentration, 200 groups of experimental data were collected for analysis. For the collected PA signal waveform, we used a 10 kHz–5 MHz bandpass filter for preliminary processing to extract an effective signal. Then, these signals were superimposed and averaged on the amplitude to obtain a representative waveform.  Figure 2 shows the difference in waveform when the concentration is increased from 0 mg/dL to 10,000 mg/dL. It can be seen from the figure that the waveform of the PA signal, which is excited by the same structure, is basically the same, while there is a slight difference in amplitude. The higher the concentration of the glucose, the higher the signal amplitude.

In order to clarify the effect of glucose concentration on the PA signal, we selected the peak-to-peak value and waveform energy feature in the waveform to quantify the linear relationship. Figures 2(a) and 2(c) show the relationship between these features and glucose concentration. The black dots in the figure record the distribution range of 200 groups of data at each concentration, and the red star is the representative waveform, which is the superimposed average of those data. In the case of a higher-concentration change, those features have a good linear relationship, and the correlation coefficient *R*^2^ has reached more than 0.9. The waveform energy feature has a better linearity, and the *R*^2^ is above 0.99.

**Figure 3:**
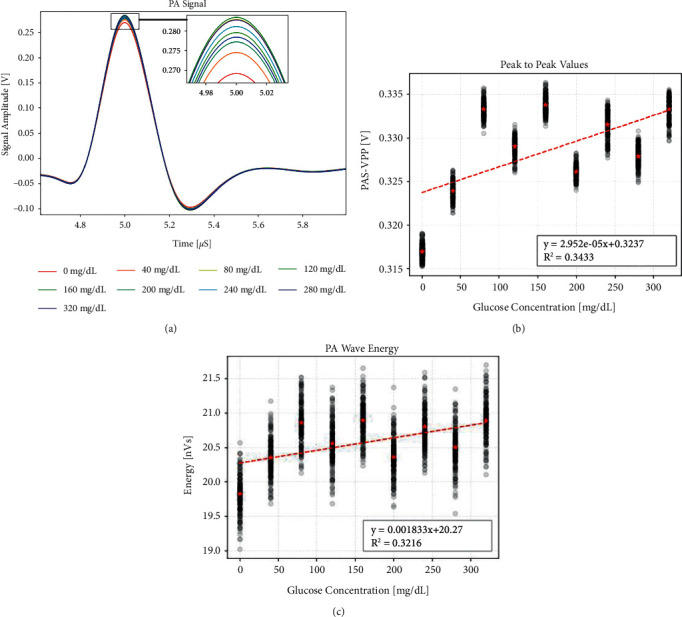
Photoacoustic signal difference in low-concentration glucose solution. (a) Waveform difference. (b) Peak-to-peak difference. (c) Waveform energy difference.

However, the normal blood glucose level of the human body is between 3.9 and 6.1 mmol/L, that is, within the range of 70.2–109.8 mg/dL. The concentration of 10,000 mg/dL is far beyond this range. For this reason, we repeated the experiment with a gradient of 20 mg/dL and obtained the changes of PA signal when the glucose concentration varied in the range of 0–360 mg/dL. The waveform, peak-to-peak values, and energy features are shown in Figure 3. It can be seen from the figure that there is no obvious linear relationship between the glucose concentration and the PA signal. At high concentrations, each group of solutions has a certain deviation from the fitted straight line, as shown in Figure 2, which indicates a high level of external interference, so this result is taken for granted. In low-concentration experiments, such external interference is far greater than the change caused by concentration, so we cannot directly obtain a clear linear relationship.

### 4.2. Study of External Interference

In this experimental system, the external factors that affect the amplitude of the PA signal mainly include laser energy, liquid temperature, and the distance between the liquid surface and the transducer probe. In order to clarify how these external factors will affect the results of the experiment, we explore the influence of these factors.

**Figure 4:**
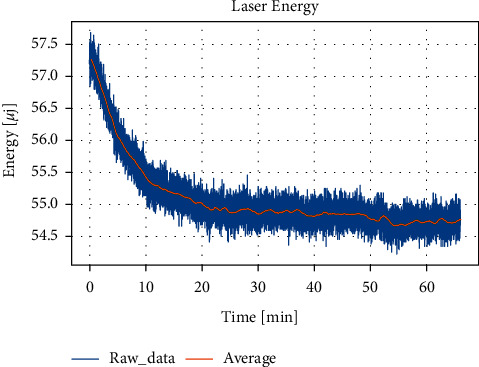
Fluctuation of laser energy over time. raw_data is the original data, and average is the moving average of 200 groups of data.

**Figure 5:**
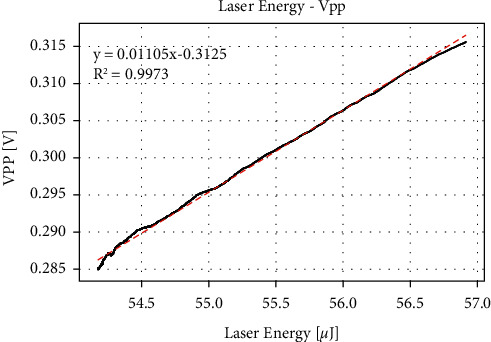
Peak-to-peak value changes with the laser energy.

We use a spectroscope to split a part of the laser to the energy meter to record the energy change during the experiment.  Figure 4 shows the change of laser energy measured over time. Due to its large fluctuation, we take the moving average with 200 data points. It can be seen that the laser energy showed a decreasing trend in the first 20 minutes and then stabilized, but there were still fluctuations after 20 minutes. Although the data was measured after the laser worked for 20 minutes in the experiment, the subsequent fluctuations could not be avoided.  Figure 5 shows a linear relationship between the peak-to-peak value of the PA signal and the laser energy. Each increase of 1 *μ*J of laser energy can induce a peak-to-peak change of about 11.05 mV. From Figure 2(b), it can be calculated that to change the peak-to-peak value by 1 mV, a change of concentration of 188 mg/dL is required. Therefore, the error caused by the energy fluctuation of 0.01 *μ*J is equivalent to the effect of the concentration change of 20.8 mg/dL. This shows that subsequent fluctuations cannot be ignored.

**Figure 6:**
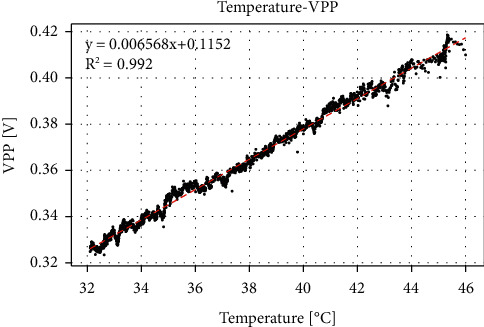
Peak-to-peak value changes with temperature.

Laser irradiation will have a certain heating effect on the water surface, and the sample temperature will not necessarily remain constant, so the solution temperature is one of the variables that are difficult to control. Therefore, we explored the effect of temperature changes on the PA signal. In the experiment, we heated the 200 mg/dL glucose solution and injected it into the PA cell, and when it cooled to 46°C, we started to record data.  Figure 6 shows the relationship between peak-to-peak value of PA signal and liquid temperature. It shows that there is a certain linear relationship between temperature and peak-to-peak value, and temperature changes by 1°C; the peak-to-peak value changes by about 6.57 mV. Therefore, the error caused by the temperature fluctuation of 0.01°C is equivalent to the effect of the concentration change of 12.35 mg/dL. This shows that the error caused by temperature is very large.

**Figure 7:**
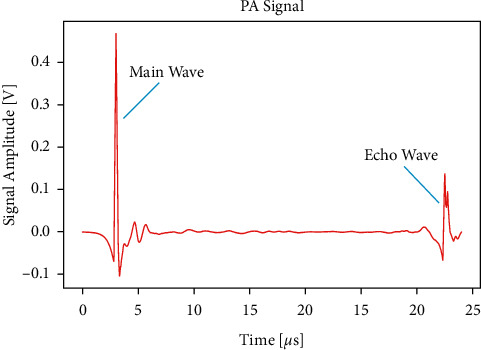
Main PA signal and echo signal.

**Figure 8:**
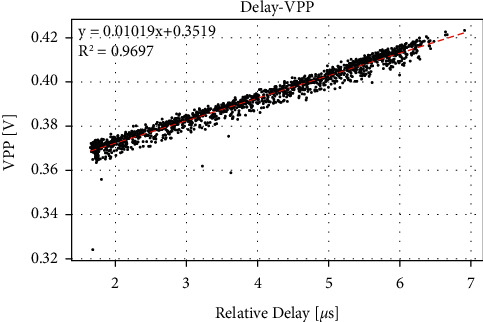
The relationship between peak-to-peak value and delay in different surface distance.

In the experiment, when the solution was replaced, there was a little residual liquid on the wall of the container, and when 25 mL of liquid was manually measured, there was a more or less deviation in the volume. As a result, in each experiment, there is a certain deviation in the distance between the liquid surface and the ultrasonic probe, which slightly misaligned the position where the PA signal is generated and the position where the ultrasound probe is focused, thereby affecting the collected PA signal. We also verify the relationship between them through experiments. Since the bottom of the PA cell is airtight, the PA signal will be reflected back after reaching the ultrasonic probe and then reflected back again after reaching the water surface. This makes it possible to receive an echo signal within 30 *μ*s after receiving the main PA signal, as shown in Figure 7. The interval between these two peaks is the time required for sound to travel from the surface to the ultrasonic probe and come back. This kind of time-lapse signal is called delay signal. Although we cannot directly measure the distance from the liquid surface to the ultrasonic probe, based on the delay signal, we can indirectly obtain the distance by measuring the time delay and then analyze its influence on the PA signal. Firstly, we used distilled water for the experiment. In the experiment, the change of surface distance was increased to more than 1 cm, making it exceed the influence of temperature on sound velocity. We use a syringe to inject or withdraw a random volume of liquid from the PA cell, while the range of volume variation is not more than 5 mL.  Figure 8 shows the relationship between peak-to-peak value and relative time delay. It can be seen from the figure that they have a significant linear relationship, and each time the delay increases by 1 *μ*s, the peak-to-peak value increases by 10.19 mV. The difference of around 6 *μ*s between the maximum and minimum of delay is caused by the change of the liquid volume of 5 mL. On average, if the volume changes by 0.01 mL, it can cause a peak-to-peak change of 0.122 mV, which is equivalent to the effect of the concentration change of 23.0 mg/dL. It can be seen that the change of surface distance also has a large influence.

**Figure 9:**
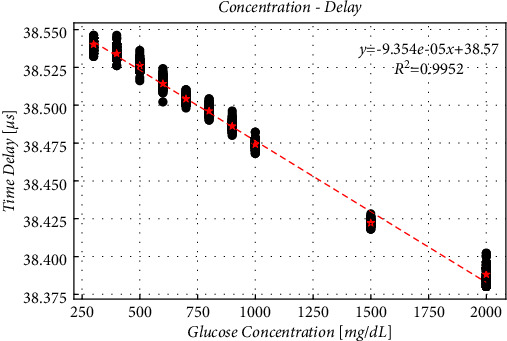
Delay in different concentrations at the same surface distance.

In addition, it is not only the surface distance that affects the delay, but also the sound velocity, and the velocity is different in different concentrations of glucose. We noticed the experiment of Dasa et al. [30]. The laser is irradiated upward from the bottom of the transparent beaker, and the ultrasound probe is located at a fixed position above the bottom. In this way, the position of the probe and the signal source is changeless. Therefore, it is possible to study the relationship between the delay and different concentrations at the same distance. We got Figure 9 in this way. It shows the linear relationship between glucose concentration and time delay. And when the concentration changes by 1 mg/dL, the delay changes by 9.35 *∗* 10^−5 *μ*s. This means that 1 *μ*s of delay variation equivalent of 1.07 *∗* 10^4 mg/dL change in concentration, which is much larger than that used in the experiment. Therefore, compared with the influence from surface distance, the change of sound velocity in different concentration can be ignored.

### 4.3. Calibration

**Figure 10:**
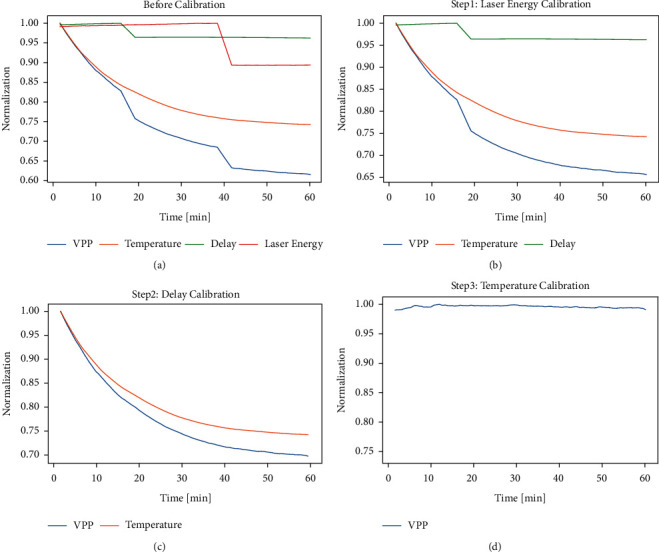
Laser energy, peak-to-peak value, delay, and temperature changes with time in the experiment and correction process. (a) Before calibration. (b) Calibration by laser energy. (c) Calibration by delay. (d) Calibration by temperature.

From the above analysis, it can be seen that external factors, such as laser energy, temperature, and liquid level, will have large influence on peak-to-peak value.  Figure 10(a) shows the variation of peak-to-peak value and delay of 200 mg/dL glucose solution in 1 hour. The liquid temperature and the laser energy were also recorded. For easy observation, we calculated the moving average by 200 data points. As shown in the figure, in the experiment at 20 minutes, about 1 mL of liquid was artificially removed from the PA cell to make the liquid level drop significantly. It can be seen that there is a very obvious decrease in delay, as well as the peak-to-peak value. At 40 minutes, the laser energy was artificially decreased, which also resulted in a significant change in peak-to-peak value. The experiment was carried out at room temperature. As there was no temperature control, the liquid temperature dropped by about 8°C within 1 hour.

Based on the analysis in Section 4.2, the laser energy, temperature, delay, and so on are all proportional to the peak-to-peak value, so the following methods can be used to correct the peak-to-peak value at a single concentration:

(4) $$\begin{array}{c} y_{Vpp}=x_{Vpp}\cdot \frac{1}{x_{LE}+b_{LE}}\cdot \frac{1}{x_{D}+b_{D}}\cdot \frac{1}{x_{T}+b_{T}}\text{.} \end{array}$$

*y*_vpp_ is peak-to-peak value after calibration, *x*_vpp_ is peak-to-peak value before calibration, *x*_LE_ is the corresponding laser energy, *b*_LE_ is the correction intercept of laser energy, *x*_D_ is delay, and *x*_T_ is temperature. The calibration can be made by adjusting the value of parameter *b*, as shown in Figures 10(b)∼10(d).

It is calculated that when *b*_LE_, *b*_D_, and *b*_T_ are set to specific values, the original fluctuations caused by the change of laser energy, temperature, and liquid height can be eliminated. This keeps the corrected peak-to-peak value curves flat. Comparing Figures 10(a) and 10(d), it can be seen that, after calibration, the variation of the peak-to-peak value is reduced from 40% to 1%.

**Figure 11:**
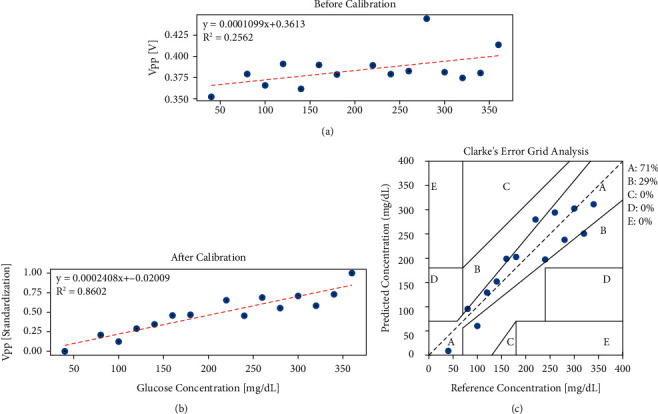
Relationship between the peak-to-peak values and glucose concentration before and after calibration and the Clarke's error grid analysis. (a) Before calibration. (b) The relationship after calibration. (c) Clarke's error grid analysis after calibration.

As for glucose solutions at different concentrations, the sound velocity is different, which results in changes of the correction intercept *b*. But, according to the analysis in Figure 9, the delay caused by sound velocity can be ignored. Zhao et al. studied the relationship between the temperature and sound velocity at different solution concentrations [23]. They found that the influence of temperature in different concentrations is almost the same. Therefore, we can use the same correction intercept to calibrate different concentrations of glucose.  Figure 11 shows the result of calibration in above method. It shows that the original irregular data has a strong linear relationship after correction, and *R*^2^ has been increased from 0.26 to 0.86. Moreover, as long as the light path and the volume of the liquid do not change, the premeasured correction intercept *b* can always be used for calibration. According to the fitting line in Figure 11(b), we can calculate the glucose concentration backwards by calibrated peak-to-peak value. In this way, we got the Clarke's Error Grid (CEG), shown as Figure 11(c).

## 5. Discussion

It can be seen from the high-concentration experiment that the PA signal extracted by the device used in this paper did show a linear relationship with the concentration of glucose solution. And at high concentration, even without any calibration measures, the linearity of waveform energy and solution concentration could also reach more than 0.99. This is because the changes caused by glucose concentration are far greater than the influence of the external environment. However, when the concentration was close to the human body level, the deviation caused by external factors was very obvious, and it must be further calibrated.

As shown in Figure 11, the linearity could get 0.8 even in the low-concentration experiment after calibration. This indicates that the influence from the external environment is measurable and can be decreased by the correction method. Moreover, the result was not individual or inimitable. The same correction intercept can be used in different times of experiment and the linearity can all improve more or less. However, if the structures of equipment are changed, the original correction intercept will not be used anymore and the recalculated intercept is required. In addition, the measurement of external influence could not be so highly accurate; thus, there still were some errors in the result after calibration. The more precise the measurement, the better result may be got.

In the structure used in this paper, the ultrasonic transducer was immersed in the liquid, and the PA signal was generated and propagates directly in the liquid. Compared with air, the attenuation of ultrasonic signal in liquid was very small, so the device could ensure good signal fidelity. Pleitez had designed a PA cell that amplified the PA signal through resonance and reduced the influence of air humidity [12]. However, in our device, the PA signal excited by the single-pulse laser was not weak, and the transmission attenuation was small. The final signal detected by the ultrasonic transducer had a good signal-to-noise ratio, so there was no need for the PA cell to provide resonance amplification.

It is found in the experiment that the height of the liquid would have an influence on the signal. This is because the PA signal is generated at the air–liquid interface, and the ultrasonic probe is focused type. Changes in liquid height can make the signal source closer to or away from the focus, resulting in inconsistent energy reception. And there was a bias in the volume of the artificially measured solution when changing liquid. Fixing the probe on the top and irradiating laser from the bottom of beaker is a good way to avoid this problem. However, when the laser hits the bottom of the beaker, it will produce strong oscillations which complicate the PA signal. So, we adopted the original structure that calibrated by delay.

The calibrated data was much better than raw data, but fluctuation still remains. No matter the waveform, temperature, or laser energy, there will be errors in the measurement. These errors will be amplified during calibration. We must average multiple data to reduce the error. In order to get more accurate data, more precise measurement is necessary in the future.

## 6. Conclusion

The photoacoustic signal, which is excited by a single pulse laser that directly irradiated on the surface of the liquid, does have a certain correlation with the concentration of the glucose solution. This correlation is very obvious at higher concentration changes. When the concentration is close to the human body level, external influences such as laser energy fluctuations, temperature changes, and small displacements of structural positions cannot be ignored. And using multiple external variables for calibration can improve the linearity. In the future, more accurate temperature monitoring and more repeatable sampling structures will be needed to make better use of photoacoustic effects in noninvasive blood glucose detection.

## Data Availability

The research data used to support the findings of this study are available from the corresponding author upon request.
